# Understanding the effects of ethanol on the liposome bilayer structure using microfluidic-based time-resolved small-angle X-ray scattering and molecular dynamics simulations†

**DOI:** 10.1039/d3na01073b

**Published:** 2024-03-25

**Authors:** Masatoshi Maeki, Niko Kimura, Yuto Okada, Kazuki Shimizu, Kana Shibata, Yusuke Miyazaki, Akihiko Ishida, Kento Yonezawa, Nobutaka Shimizu, Wataru Shinoda, Manabu Tokeshi

**Affiliations:** a Division of Applied Chemistry, Faculty of Engineering, Hokkaido University Kita 13 Nishi 8, Kita-ku Sapporo 060-8628 Japan m.maeki@eng.hokudai.ac.jp tokeshi@eng.hokudai.ac.jp +81-11-706-6745 +81-11-706-6773 +81-11-706-6744; b JST PRESTO 4-1-8 Honcho, Kawaguchi Saitama 332-0012 Japan; c Institute of Materials Structure Science, High Energy Accelerator Research Organization (KEK) Tsukuba Ibaraki 305-0801 Japan; d Graduate School of Chemical Sciences and Engineering, Hokkaido University Kita 13 Nishi 8, Kita-ku Sapporo 060-8628 Japan; e Department of Materials Chemistry, Nagoya University Chikusa-ku Nagoya 464-8603 Japan; f Research Institute for Interdisciplinary Science, Okayama University Okayama 700-8530 Japan

## Abstract

Lipid nanoparticles (LNPs) are essential carrier particles in drug delivery systems, particularly in ribonucleic acid delivery. In preparing lipid-based nanoparticles, microfluidic-based ethanol injection may produce precisely size-controlled nanoparticles. Ethanol is critical in LNP formation and post-treatment processes and affects liposome size, structure, lamellarity, and drug-loading efficiency. However, the effects of time-dependent changes in the ethanol concentration on the structural dynamics of liposomes are not clearly understood. Herein, we investigated ethanol-induced lipid bilayer changes in liposomes on a time scale from microseconds to tens of seconds using a microfluidic-based small-angle X-ray scattering (SAXS) measurement system coupled with molecular dynamics (MD) simulations. The time-resolved SAXS measurement system revealed that single unilamellar liposomes were converted to multilamellar liposomes within 0.8 s of contact with ethanol, and the *d*-spacing was decreased from 6.1 (w/o ethanol) to 4.4 nm (80% ethanol) with increasing ethanol concentration. We conducted 1 μs MD simulations to understand the molecular-level structural changes in the liposomes. The MD simulations revealed that the changes in the lamellar structure caused by ethanol at the molecular level could explain the structural changes in the liposomes observed *via* time-resolved SAXS. Therefore, the post-treatment process to remove residual ethanol is critical in liposome formation.

## Abstract 2

We investigated ethanol-induced structural changes in liposomes on a time scale from microseconds to tens of seconds using a microfluidic-based small-angle X-ray scattering (SAXS) measurement system coupled with molecular dynamics (MD) simulations.

## Graphical abstract

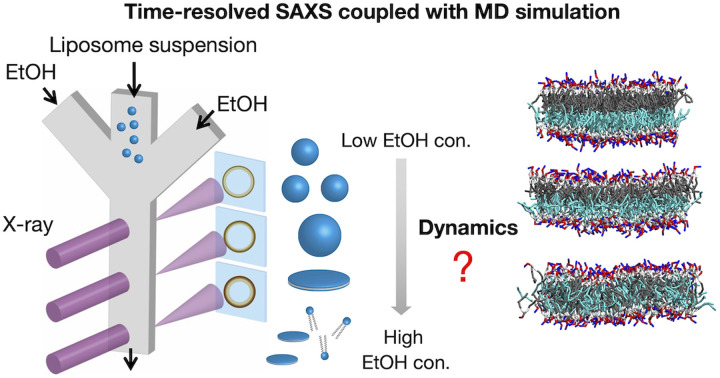

## Introduction

The recent progress in ribonucleic acid (RNA) therapy is attracting attention in the fields of personalized nanomedicine and intractable and infectious disease treatments.^1–3^ RNA is unstable and should be delivered to target tissues without RNase degradation, and thus, RNA delivery systems are indispensable in ensuring therapeutic effects. Lipid-based nanoparticles, such as lipid nanoparticles (LNPs) and liposomes, are promising carriers for RNA delivery to target organs.^4^ In 2020, Moderna and Pfizer-BioNTech COVID-19 vaccines, which encapsulate messenger RNAs (mRNAs) within LNPs, were approved worldwide and could protect against severe COVID-19.^5,6^

Generally, lipid-based nanoparticles, including LNPs and liposomes, are prepared using various methods, including film hydration, ethanol injection, and detergent removal.^7^ After particle preparation, the sizes of the nanoparticles are tuned *via* sonication or extrusion. Recently, microfluidic-based ethanol injection has been widely used in producing size-controlled nanoparticles without complicated size-tuning processes.^8–15^ In this method, aqueous and lipid-containing ethanol solutions are introduced into a microfluidic device, and the ethanol is then continuously diluted with the aqueous solution within the device. Lipid molecules aggregate upon ethanol dilution, followed by nanoparticle formation, and thus, ethanol is critical in nanoparticle formation in terms of nanoparticle size, structural change, and drug loading. However, the effects of time-dependent changes in ethanol concentration on lipid-based nanoparticles remain unclear owing to the lack of *in situ* measurement methods.

In microfluidic-based liposome preparation, ethanol affects liposome size and the bilayer structural changes during liposome formation and dialysis. We reported the effects of the flow conditions and microchannel structure on the liposome size.^14,16,17^ Generally, large liposomes form at a slow ethanol dilution rate, whereas small liposomes form at a rapid ethanol dilution rate. Additionally, the liposome suspension prepared using the microfluidic device contains ethanol, and residual ethanol may induce fusion and structural changes within the liposomes.^18^ Understanding the effects of time-dependent changes in ethanol concentration on liposome formation and ethanol removal processes, such as dialysis, enables the development of novel microfluidic devices for more precise size and structural control, including lamellarity and higher drug loading efficiency.^19–21^

Transmission electron microscopy (TEM), nuclear magnetic resonance (NMR) spectroscopy, and small-angle X-ray scattering (SAXS) are generally used to determine the liposome structure under static conditions, but they lack the temporal resolution to determine the changes in the liposome structure.^22–26^ The measurement of structural changes in liposomes with time-dependent changes in ethanol concentration, in particular, is a major challenge in the fields of nanomaterial science, structural biology, and analytical chemistry. To understand the effects of time-dependent changes in ethanol concentration on the structural changes in liposomes, time-resolved SAXS using a rapid stopped-flow mixing technique or microfluidic devices is a suitable measurement method. Angelov *et al.* reported the structural changes in plasmid deoxyribonucleic acid (DNA) and lipid nanocarriers within 11–95 ms,^27^ and Ghazal *et al.* used microfluidic-based SAXS in the continuous characterization of multilamellar vesicles.^23^ Although these devices and techniques enable time-resolved SAXS, the lipid bilayer changes in liposomes, such as the ethanol-induced conversion of single unilamellar vesicles to multilamellar vesicles (MLVs), remain unclear.

Herein, we investigated the ethanol-induced lipid bilayer changes in liposomes over microseconds to tens of seconds using a microfluidic-based SAXS measurement system coupled with molecular dynamics (MD) simulations to provide a plausible detailed structural model of the lamellar structure detected using SAXS. The microfluidic-based SAXS measurement system may capture the structural variations in the liposomes induced by time-dependent changes in ethanol concentration. We confirmed the time-dependent formation of a multilamellar structure and a change in the lipid bilayer distance in the presence of a low concentration of ethanol. MD simulations within 1 μs microscopically complemented the MLV structures at different ethanol concentrations. Based on both approaches, 50% ethanol was a critical concentration to form liposomes. The combination of time-resolved SAXS and MD simulations enabled us to understand the structural changes in liposomes in detail.

## Results and discussion

### Development of a SAXS measurement system based on a computational fluid dynamics (CFD) simulation

**Fig. 1:**
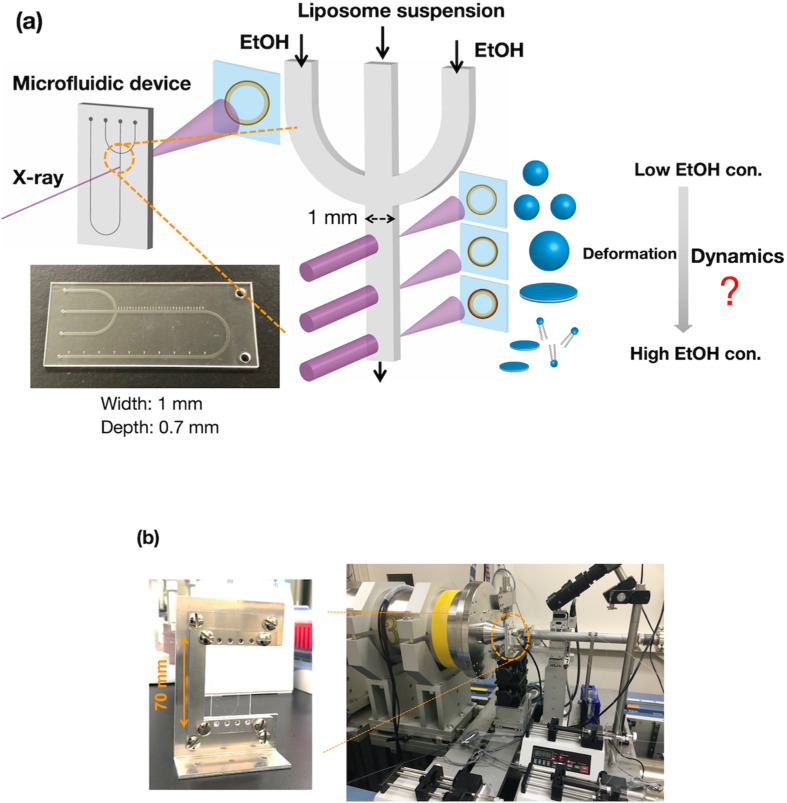
(a) Schematic diagram of the time-resolved SAXS measurement system with a microfluidic device. (b) Images of the measurement system at beamline BL15A2 at the Photon Factory. The microchannel is 1 mm wide and 0.7 mm deep. EtOH, ethanol; con., concentration.

To evaluate the effect of ethanol on the liposome bilayer, the ethanol concentration in the liposome suspension should be gradually increased, depending on the measurement time, and thus, the use of a typical stopped-flow system is unsuitable. In addition, the time scale of liposome formation is nanoseconds and conventional SAXS does not exhibit a sufficient temporal resolution. Therefore, we measured and simulated the changes in the liposome bilayer structure during the reverse process of liposome formation, *i.e.*, liposome deformation, with a gradual increase in ethanol concentration. The time-resolved microfluidic-based SAXS measurement system, with a flow-focusing microchannel, is shown in Fig. 1.^23,28^ To measure the structural dynamics of the liposomes using ethanol, the ethanol concentration in the liposome suspension should be gradually increased, depending on the residence time. Therefore, instead of using a micromixer typically employed in liposome production, we utilized a flow-focusing type microfluidic device for SAXS measurements. In the microchannel, ethanol molecules are transported from the side-flow stream to the center stream *via* Fick's law of diffusion. Ethanol induces structural changes in the liposomes, depending on the increase in the ethanol concentration. The flow conditions and dimensions of the microchannel affect the ethanol diffusion behavior and signal-to-noise (*S*/*N*) ratio of the X-ray scattering intensity, and thus, we performed preliminary SAXS to optimize the flow conditions and microchannel structure. A total flow rate of 50 μL min^−1^ and a flow rate ratio (FRR) of the side channels to the center channel of 5 are suitable for observing the changes in liposome structure with a high *S*/*N* ratio. The dimensions of the microchannel are optimized to be 1 mm wide and 0.7 mm deep.

**Fig. 2:**
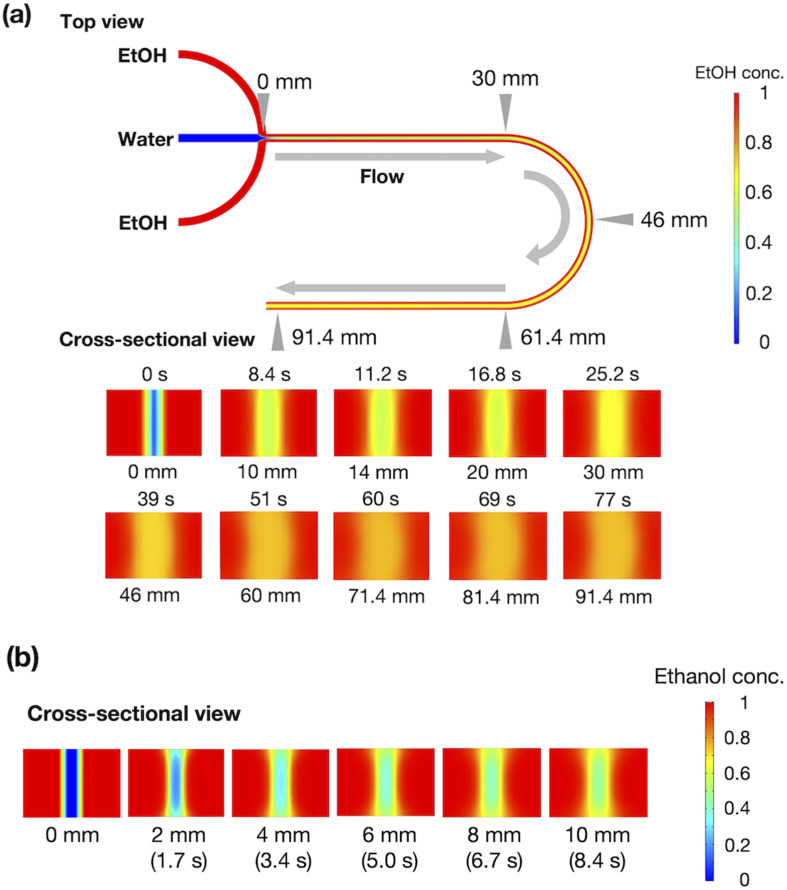
(a) CFD simulation of ethanol concentration within the microchannel. (b) Ethanol concentration in the cross-section at 0–10 mm. The red and blue colors represent ethanol and water, respectively.

Fig. 2(a) shows the results of the CFD simulation of the ethanol concentration within the microchannel. Under the flow conditions, the ethanol concentration reaches 83% when the ethanol and aqueous phases are completely mixed. As shown in the cross-sectional views, the ethanol concentration in the center stream reaches almost 80% in 39 s. Fig. 2(b) shows the CFD-simulated cross sections from 0 to 10 mm after mixing the ethanol and aqueous solutions. At the 2 mm position, ethanol does not diffuse into the center stream, and the ethanol concentration in most regions of the liposome suspension is <30%. The ethanol concentration in the liposome suspension stream reaches almost 50% to 65% at the 10 mm position. These results suggest that the microfluidic SAXS system may yield data regarding the ethanol-induced changes in the liposome structure.

### Characterization of the liposome under static conditions

**Fig. 3:**
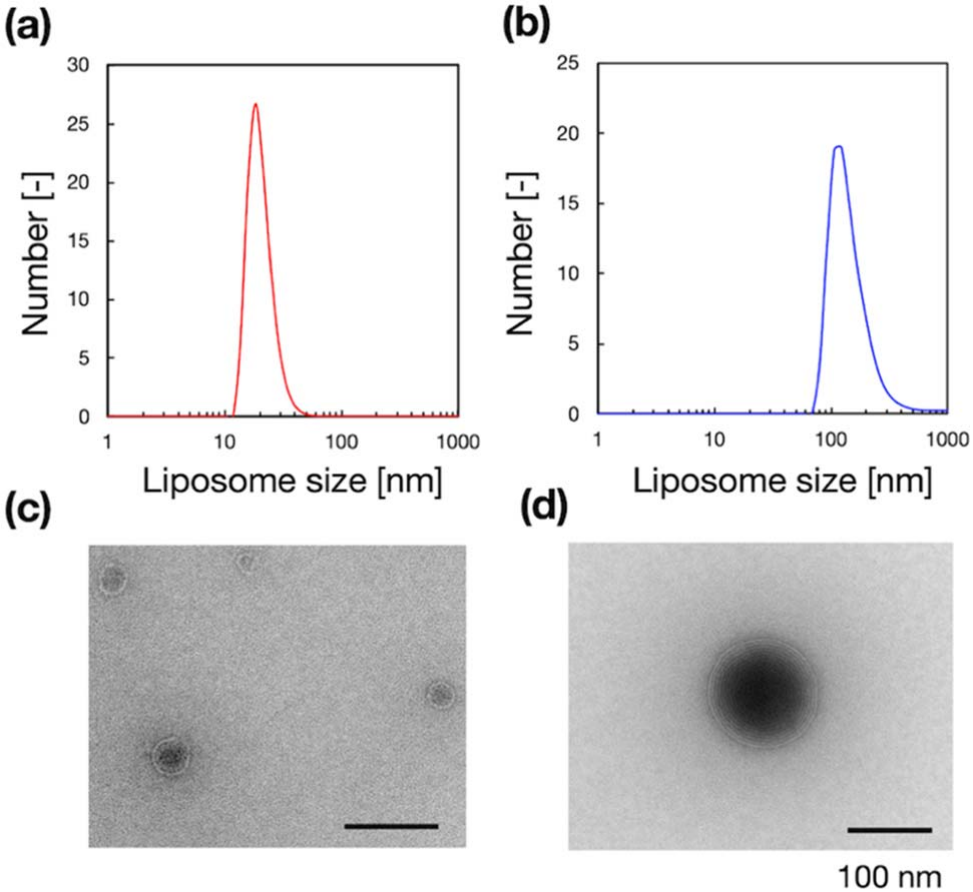
(a and b) Number-weighted liposome size distributions and (c and d) TEM images of the liposomes, which were produced using the microfluidic device (a and c) or conventional hydration (b and d).

To validate the microfluidic SAXS system, we studied 1-palmitoyl-2-oleoyl-*sn*-glycero-3-phosphocholine (POPC)-based liposomes under static conditions. The liposomes were prepared using two different methods: conventional hydration and the microfluidic method. The ethanol dilution rate plays a significant role in controlling the size of LNPs. Small and homogeneous-sized liposomes are formed through rapid ethanol dilution using microfluidic devices. Conversely, slow ethanol dilution leads to the formation of large and heterogeneous liposomes. Consequently, we anticipate that the preparation method influences the inner structure of liposomes. Fig. 3(a) and (b) show the liposome size distributions, as determined *via* dynamic light scattering (DLS). The sizes of the liposomes prepared using the microfluidic and hydration methods are 20 and 160 nm, respectively. The liposomes prepared using the microfluidic device are theoretically smaller and should display unilamellar structures. Conversely, the large liposomes prepared *via* hydration form multilamellar structures rather than single unilamellar structures. To confirm the liposome structures, we performed TEM, and Fig. 3(c) and (d) show the TEM images of the liposomes. The results of TEM correspond to those of DLS, and the liposomes form different structures, depending on their sizes and the preparation method. The liposomes prepared using the microfluidic device exhibit 20 nm-sized unilamellar structures, and the 160 nm-sized liposomes prepared *via* hydration display multilamellar structures.

**Fig. 4:**
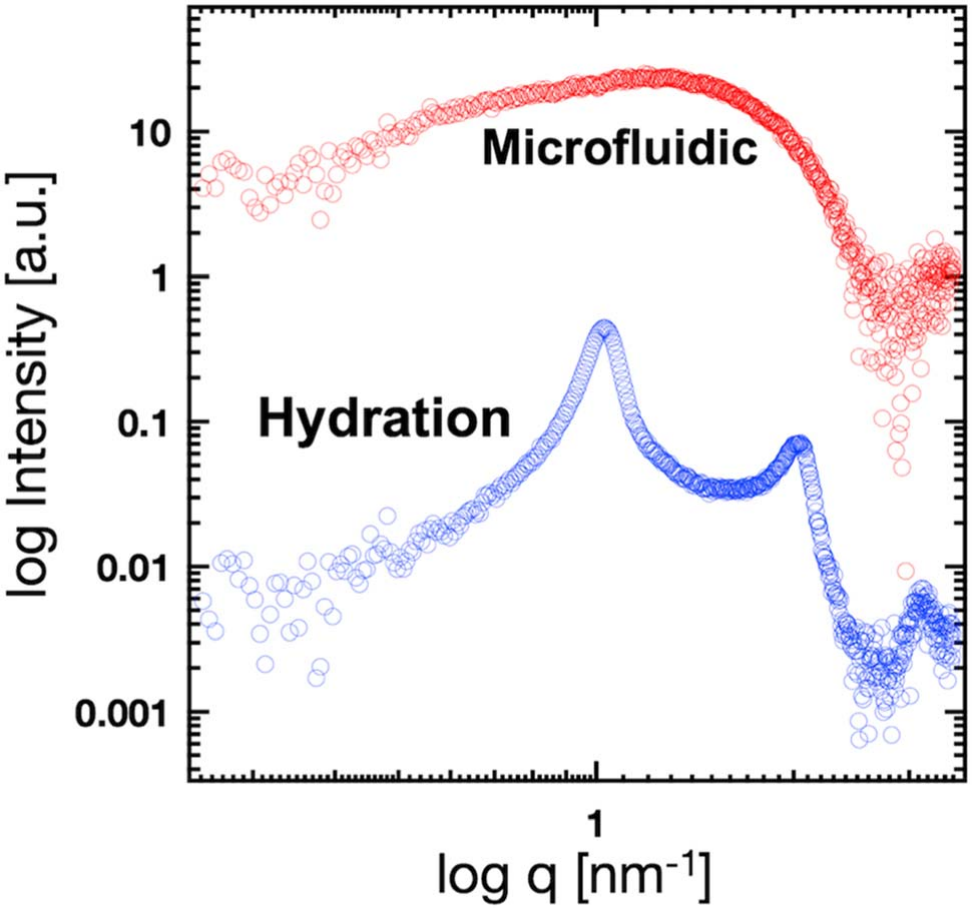
SAXS profiles of the liposomes prepared using the microfluidic device (red circles) or hydration (blue circles).

We used these liposome sizes to validate the microfluidic SAXS measurement system. Fig. 4 shows the SAXS profiles of the liposomes prepared using the microfluidic device (20 nm, red circles) or hydration (160 nm, blue circles). In the profile of the 20 nm-sized unilamellar liposomes, we do not observe a notable SAXS peak caused by the repeated multilamellar structure, and the SAXS profile indicates the spherical shapes of the liposomes. In contrast, the SAXS profile of the 160 nm-sized liposomes suggests the formation of multilamellar structures. We confirm the Bragg peaks at 1.03, 2.05, and 3.12 nm^−1^, corresponding to the ordered lipid bilayer membranes of the multilamellar liposomes.^29^ Based on the Bragg peaks, the lamellar *d*-spacing is 6.1 nm, which is the typical value for liposomes, and the thickness of two POPC bilayers is 4.21 nm, according to previous studies.^30^ Based on these results, the thicknesses of the hydration layers of the POPC-based liposomes are approximately 1.9 nm. Hence, the microfluidic SAXS system enables the determination of the liposome structure and discrimination between the uni- and multilamellar structures.

### SAXS and the ethanol-induced changes in the liposome bilayer structure

**Fig. 5:**
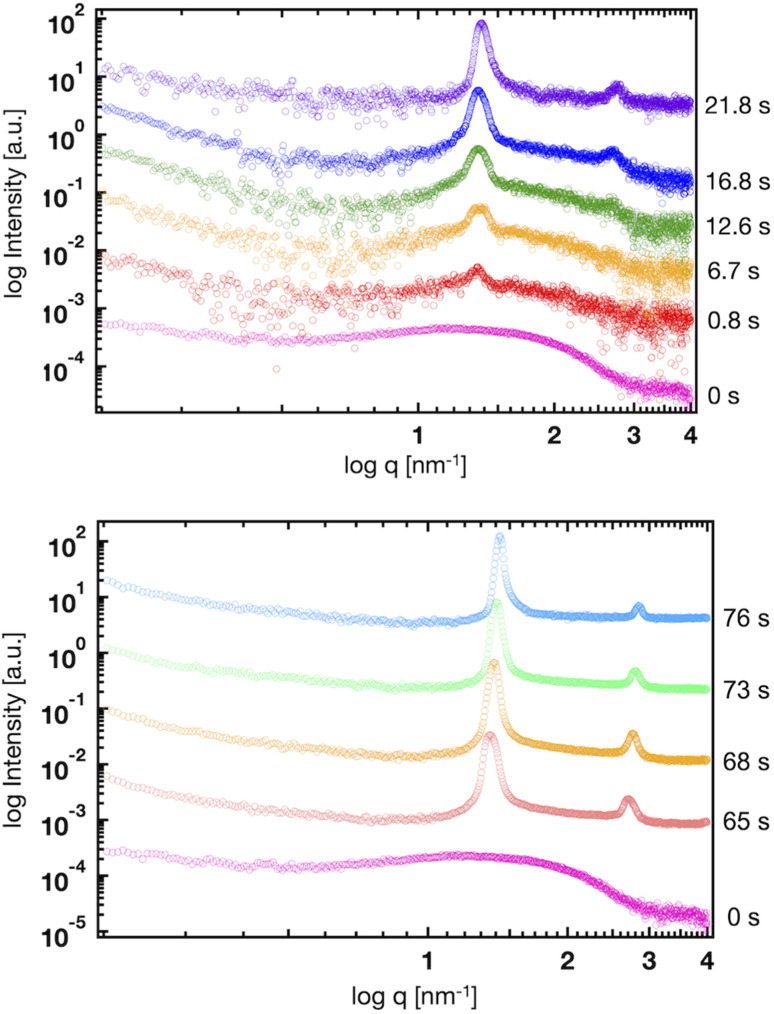
Time-resolved SAXS profiles of the liposomes at contact times of 0, 0.8, 6.7, 12.6, 16.8, 21.8, 65, 68, 73, and 76 s.

Fig. 5(a) shows the SAXS profiles of liposomes with a time-dependent increase in the ethanol concentration. The liposome suspension was introduced into the microfluidic device and ethanol was introduced from the side microchannels. To obtain each SAXS profile, the measurement positions were shifted along the microchannel. The Bragg peak at 1.35 nm^−1^ is observed within 1 s after contact with ethanol. The intensities of the Bragg peaks gradually increase with increasing contact time, and the secondary Bragg peak is observed at 2.70 nm^−1^ after a contact time of 12.6 s. Remarkably, the Bragg peak is shifted from 1.03 to 1.35 nm^−1^*via* contact with ethanol for <1 s (Fig. 4 and 5), and thus, ethanol reduces the *d*-spacing by 1.4 nm (6.1 to 4.7 nm). In addition, the first and second Bragg peaks are shifted to a wide-angle region with increasing ethanol concentration. During the contact time from 0.8 to 76 s, the first Bragg peak is shifted from 1.35 to 1.43 nm^−1^, suggesting that the *d*-spacing is reduced from 4.7 to 4.4 nm.

Based on the CFD study, the ethanol concentration at the center of the liposome suspension stream reaches 20–30% within 1.7 s, although it reaches 60–70% at the interface between the liposome suspension and ethanol stream. The ethanol concentration at the center of the liposome suspension stream is maintained at 70% within a residence time of 25 s (Fig. 2). In this microfluidic-based SAXS measurement, the SAXS profile represents the average liposome structure at each measurement point. Therefore, most liposomes display single unilamellar structures at a residence time of 0.8 s, and a few liposomes at the interface between the liposome suspension and ethanol exhibit multilamellar structures. Ethanol immediately induces changes in the liposome structure from single unilamellar to multilamellar after contact with the liposome suspension of <0.8 s. In addition, the 1.4 nm reduction in *d*-spacing due to contact with ethanol for <0.8 s may suggest the significance of SAXS measurements at shorter ethanol contact times. Ethanol may change the liposome structure over a few microseconds to tens or hundreds of microseconds and induce a reduction in *d*-spacing. This assumption is crucial in terms of liposome formation and drug encapsulation. First, the *d*-spacing should be approximately 4–5 nm from immediately after liposome formation until prior to dialysis. The *d*-spacing then increases to 6.1 nm due to the removal of ethanol. Second, the change in *d*-spacing due to ethanol affects drug encapsulation efficiency and leakage during liposome formation. A smaller *d*-spacing may lead to hydrophilic or -phobic drug leakage from the inner aqueous phase or lipid bilayer, respectively.

The microfluidic-based SAXS system enables the measurement of changes in the liposome structure within 0.8 s by increasing the flow rates of the solutions. However, obtaining SAXS data with a high *S*/*N* is challenging due to the small number of multilamellar liposomes under these measurement conditions, the small sizes of the liposomes and numbers of multilamellar layers, and the intensity of the X-ray beam. Furthermore, the high flow condition affects the ethanol concentration profile within the microchannel. To further understand the effects of ethanol on the changes in the liposome structures, we performed an MD simulation of the lamellar structure based on the results of SAXS.

### MD simulation of the lamellar structure

**Fig. 6:**
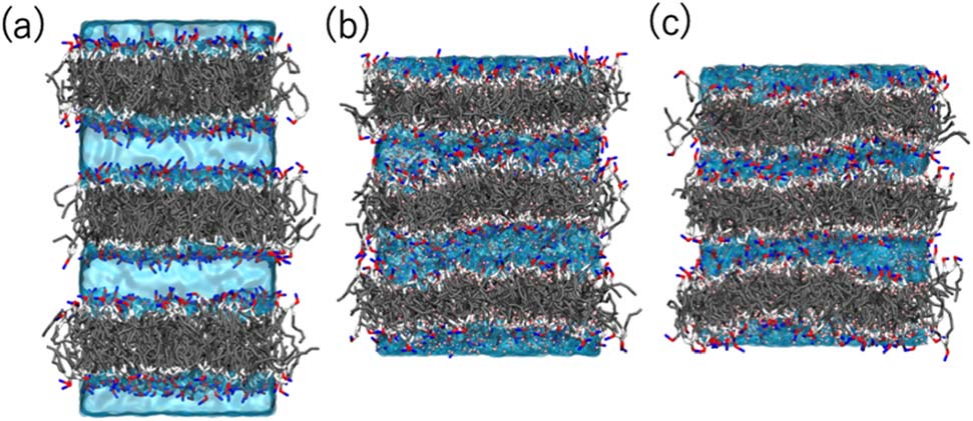
Snapshots obtained *via* the MD simulations of Systems (a) 1 (POPC bilayers in aqueous solution), (b) 2 (in 48 vol% ethanol solution), and (c) 3 (in 57 vol% ethanol solution). The POPC molecules are represented as sticks, and the water and ethanol solutions are represented by the cyan color as transparent volume. The color codes of POPC are: NC (choline): blue, PH (phosphate): red, GL (glycerol): silver, EST (ester): white, and CM, CMD2, and CT (hydrocarbons): gray.

Coarse-grained (CG) MD simulations were conducted to explore the effect of ethanol (concentration) on the POPC bilayer (lamellar) structure. First, a lamellar structure containing three POPC bilayers is constructed in the simulation box with water at a proper hydration level to explain the *d*-spacing of 6.1 nm (System 1). We calculate the structure factor using the MD trajectory to confirm the consistency of the simulation results with those obtained experimentally (Fig. S2(a)†). We then replace several randomly selected water (WAT) particles with ethanol (ETNL) particles to explore possible structural changes due to the increment of ethanol concentration, which is detected as a *d*-spacing variation (see Fig. S1† for the definitions of the CG particles). Here, we assume that one WAT particle, which represents three water molecules, is replaced by one ETNL particle, which represents a single ethanol molecule. With increasing ethanol concentration, the POPC bilayer expands laterally, reducing the membrane thickness. Thus, we may obtain reasonable POPC bilayer models (Systems 2 and 3) to explain the respective *d*-spacings of 4.7 and 4.4 nm, as observed using SAXS. The lamellar structures obtained at ethanol concentrations of 48 and 57 vol% reasonably explain the structure factors obtained *via* SAXS (Fig. S2(b) and (c)†). We conducted a 1 μs MD simulation for each system, and the snapshot obtained for each system is shown in Fig. 6. The molecular areas (cross-sectional area per lipid molecule) and *d*_PP_ (phosphate–phosphate distance along the bilayer normal, representing the membrane thickness) averaged over the MD trajectories of Systems 1–3 are shown in Table 1. With increasing ethanol concentration, the molecular area is significantly increased by 30% (System 2) and 40% (System 3), whereas *d*_PP_ is decreased by 11% (System 2) and 13% (System 3). Thus, the effective volume of the membrane is increased by increasing the ethanol concentration, because the POPC bilayer membrane is swollen with ethanol.

**Table 1:** Molecular areas and *d*_PP_ values of Systems 1–3

	Area [nm^2^]	*d*_PP_ [nm]
System 1 (water)	0.647 ± 0.005	3.85 ± 0.05
System 2 (48 vol% ethanol)	0.851 ± 0.006	3.42 ± 0.04
System 3 (57 vol% ethanol)	0.926 ± 0.009	3.34 ± 0.04

**Fig. 7:**
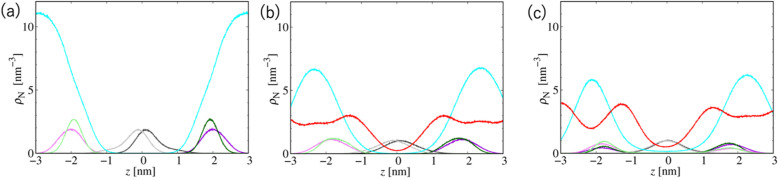
Probability density distributions of several selected CG segments along the bilayer normals in Systems (a) 1, (b) 2, and (c) 3. The line colors are WAT: cyan, ETNL: red, CT2 in the oleoyl chain: gray, PH: green, and NC: violet. The latter three lines display darker and lighter color tones in the upper and lower leaflets, respectively.

Fig. 7 shows the number density profiles of selected CG segments along the bilayer normals. In Systems 2 and 3, ethanol molecules penetrate deeply into the membrane cores, and the densities are non-zero, even at the centers of the membranes. In addition, a broader distribution of each segment in Systems 2 and 3 is observed, suggesting the larger undulations and higher flexibilities of the membranes compared to those of the membranes in System 1. The distributions of the tail segments of the oleoyl chains in the upper and lower leaflets are almost identical in Systems 2 and 3, indicating the deeper interleaflet penetration of the tail chains. Fig. 7 shows the densities of the lipids in the upper and lower leaflets separately, although in the profile of System 3, two peaks representing headgroup segments (PH and NC) may be identified. Therefore, the lipids display flip-flop motion during exchange between the upper and lower leaflets, and the flip-flop motion occurs along a transient pore through the membrane. This is observed in two bilayers of the three in System 3, and thus, the membrane swollen with ethanol at this concentration exhibits marginal stability as a bilayer. Further addition of ethanol should induce a transformation of the lamellar structure. To clarify the structural changes of the POPC bilayer with increasing ethanol concentration, a magnified view of the bilayer structure (the final snapshot from MD) is shown in Fig. S3.† The hydrocarbons are colored differently based on whether they occur in the upper or lower leaflet in the initial configuration, which reveals the lipid exchanges between the two leaflets in System 3. As shown in Fig. S3,† the interleaflet penetration of the lipid tails is clear when ethanol is added (System 2).

**Fig. 8:**
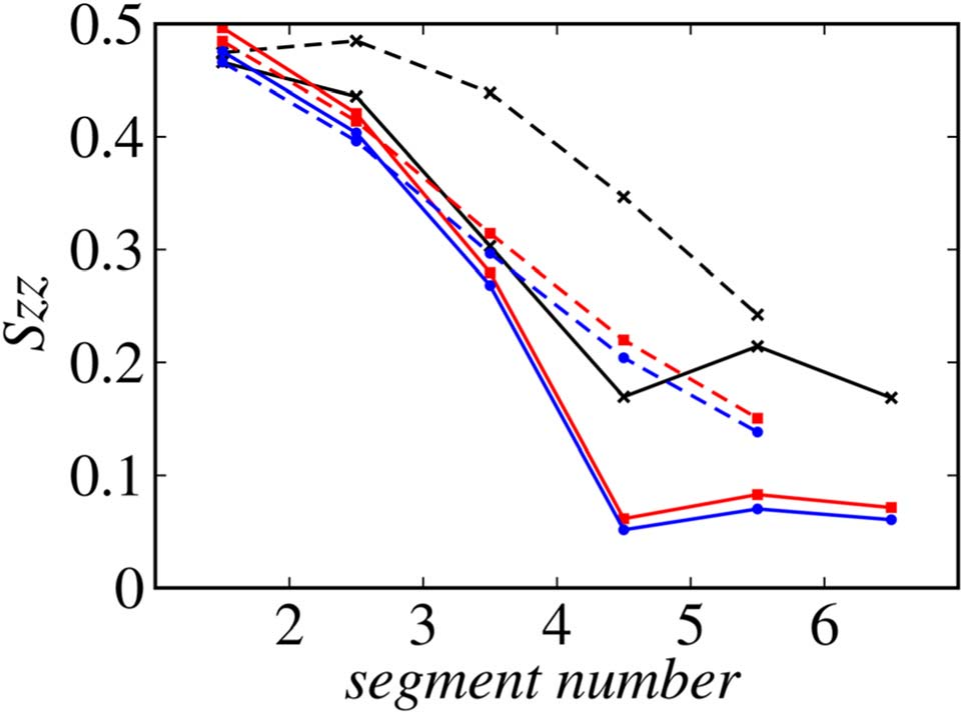
Profiles of the order along the hydrophobic chains. Solid lines: oleoyl chains and dashed lines: palmitoyl chains. The results of Systems 1, 2, and 3 are shown in black, red, and blue, respectively.

**Fig. 9:**
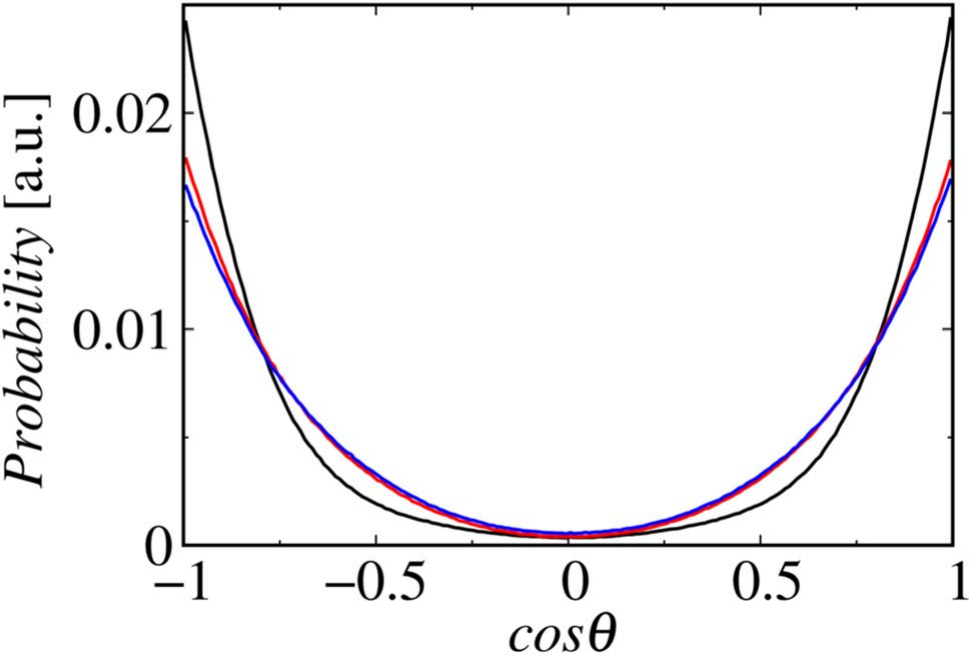
Probability distributions of the angles between the chain vectors and bilayer normals. The lines in black, red, and blue represent Systems 1, 2, and 3, respectively.

Fig. 8 shows the profiles of the segmental order along the hydrophobic chains. The segment number here represents the count starting from the EST segment to the tail CT2 segment of each chain. The results of System 1 are highly consistent with the previously reported profiles. The profile of the oleoyl chain displays a dip at the bond connecting segments 4 and 5, because segment 4 includes a *cis* double bond in the oleoyl chain, resulting in a reduction in the orientational order at this bond. The same trends are observed in the order parameters obtained *via* all-atom MD simulations.^33^ With increasing ethanol concentration, the order parameter reduces significantly, except for that at the bond of segments 1 and 2. Hence, the orientational order of the chain is significantly lowered in the membrane swollen with ethanol. Fig. 9 shows the probability distribution of the angle between the chain vector of the POPC hydrophobic chain and the bilayer normal. The chain vector is defined as the vector connecting the EST segment to the tail CT2 segment along each hydrophobic chain of POPC. Two peaks are observed at cos *θ* = 1 and −1, representing the respective orientations of the chain vectors in two different leaflets. The most likely orientations of the chain vectors are generally parallel to the bilayer normal. However, with increasing ethanol concentration, the angular distribution widens, which is consistent with the lower segmental order parameters and large molecular areas of the membranes swollen with ethanol. These structural variations at the molecular level account for the variation in the lamellar structure, as observed *via* SAXS using the microfluidic device.

## Conclusion

In this study, we investigated the effects of time-dependent changes in ethanol concentration on the bilayer structure in liposomes using time-resolved SAXS and MD simulations. The microfluidic-based SAXS measurement system could capture ethanol-induced time-dependent bilayer structural changes in liposomes. Using SAXS, we observed the structural changes in the liposomes from uni- to multilamellar within 0.8 s of contact with ethanol, and the *d*-spacing decreased from 6.1 (w/o ethanol) to 4.4 nm (80% ethanol) with increasing ethanol concentration. We also performed MD simulations to understand the bilayer structural changes in liposomes with ethanol over a shorter time. The MD simulations revealed that the changes in the lamellar structure caused by ethanol at the molecular level could explain the bilayer structural changes in the liposomes observed using time-resolved SAXS.

The temporal resolution of the SAXS system was insufficient to observe structural changes in liposomes during formation, and thus, further development of microfluidic systems combined with synchrotron facilities is required. However, our findings clarified the effects of time-dependent changes in ethanol concentration on liposome structures. Generally, the liposome suspension contains approximately 30% residual ethanol, which is removed during dialysis. Our results suggest that the post-treatment process to remove residual ethanol, in addition to the liposome formation process, is critical. Microfluidic devices focusing on the post-treatment process or integrating liposome production and post-treatment processes may accelerate the development of liposome-based nanomedicine.

## Experimental

### Materials

POPC was purchased from NOF (Tokyo, Japan), and ethanol and sodium chloride were purchased from Fujifilm Wako Pure Chemical (Osaka, Japan). Polydimethylsiloxane (PDMS, SILPOT 184 W/C) was obtained from Dow Corning Toray (Tokyo, Japan), and we purchased SU-8 3050 from Nippon Kayaku (Tokyo, Japan). Propylene glycol monomethyl ether acetate and trichloro(1*H*,1*H*,2*H*,2*H*-perfluorooctyl)silane were obtained from Sigma-Aldrich (St. Louis, MO, USA), and silicon wafers were obtained from Global Top Chemical (Tokyo, Japan). Dialysis membrane tubing with molecular weight cutoffs of 12–14 kDa was purchased from Repligen (Waltham, MA, USA).

### Fabrication of microfluidic devices

Two types of microfluidic devices were fabricated for use in liposome preparation and SAXS. For liposome preparation, we fabricated a microfluidic device *via* standard soft lithography.^16^ SU-8 was deposited onto a silicon wafer and spin-coated (MS-A100, Mikasa Shoji, Osaka, Japan) to yield an SU-8 layer with a thickness of 100 μm. We used a photomask (12 700 dpi, Unno Giken, Tokyo, Japan) designed using Adobe Illustrator CC 2017 (Adobe, San Jose, CA, USA) and a mask aligner (M-1S, Mikasa Shoji) to introduce ultraviolet (UV) light. The SU-8 mold was treated with trichloro(1*H*,1*H*,2*H*,2*H*-perfluorooctyl)silane vapor and PDMS was poured onto the molds to yield a PDMS replica. The microfluidic device was fabricated by oxygen plasma bonding using the PDMS replica and a glass slide (S1111, Matsunami Glass Ind., Ltd, Osaka, Japan). Poly(etheretherketone) (PEEK) capillaries (inner diameter = 300 μm, outer diameter = 500 μm) were connected to the inlets and the outlet of the PDMS device with super glue.

For SAXS, the microfluidic device was designed using AutoCAD 2019 (Autodesk, San Rafael, CA, USA) and fabricated using a cyclic olefin polymer (COP, Zeon, Tokyo, Japan). The microfluidic (COP) device comprised a 1 mm-thick COP substrate and 0.1 mm-thick COP film. The 1 mm-thick COP substrate was micromachined to form a microchannel, with a width and depth of 1 and 0.7 mm, respectively. The COP substrate was covered with the COP film, which were bound together *via* silane coupling.

### Preparation of liposomes

Two types of liposome suspensions were prepared using the microfluidic device or conventional hydration. The microfluidic device was used to prepare small unilamellar liposomes *via* a previously reported method.^16^ POPC was dissolved in ethanol at a concentration of 13.4 mM, and sodium chloride was dissolved in ultrapure water (Direct-Q UV system, MilliporeSigma, Burlington, MA, USA) at a concentration of 154 mM. The sodium chloride solution (saline) was filtered through a membrane filter with a pore size of 0.2 μm (Omnipore, MilliporeSigma). These solutions were respectively used to fill gastight syringes (Hamilton, Reno, NV, USA), which were connected to syringe pumps (Fusion Touch 200, ISIS, Osaka, Japan). The lipid and aqueous solutions were introduced into the microfluidic device using the syringe pumps. The liposome suspension was dialyzed against saline overnight at 4 °C. The sizes of the liposomes were measured *via* DLS using a Zetasizer Nano ZS (Malvern Panalytical, Malvern, UK). For SAXS, the liposome suspension was concentrated to 40 mM using a tangential flow filtration system (KrosFlo KR2i TFF, Repligen). The large multilamellar liposomes were prepared *via* conventional hydration. POPC was dissolved in ethanol at a concentration of 40 mM in a centrifuge tube. The ethanol was then completely evaporated under a dry N_2_ stream to form a lipid film, and the centrifuge tube was vacuumed overnight in a desiccator using a rotary pump. After the evaporation of ethanol, an appropriate amount of saline was poured into the tube, followed by mixing using a vortex mixer to yield a 40 mM liposome suspension. The liposome suspension was passed through the polycarbonate membrane filter (pore size: 200 nm) of a Mini-Extruder (Sigma-Aldrich). The sizes of the liposomes were also measured using a Zetasizer Nano ZS. The liposome suspensions prepared using the microfluidic device and conventional method were stored at 4 °C in a refrigerator prior to SAXS.

### CFD analysis

CFD analysis was conducted using COMSOL Multiphysics 5.2 (COMSOL, Burlington, MA, USA), and the details of the simulation conditions are reported in previous studies.^16^ We designed a geometric model based on the dimensions of the SAXS device, and the ethanol concentration profile was obtained using a laminar flow model (incompressible Navier-Stokes equation) coupled with the model of the transport of diluted species. The wall condition was set to the no-slip condition, and the total flow rate and FRR were set to 50 μL min^−1^ and 5, respectively. The Reynolds number is calculated to be approximately 0.7.

### Characterization of liposomes using TEM

The liposomes were characterized using TEM (H-7600, Hitachi, Tokyo, Japan) at an acceleration voltage of 100 kV. The liposome suspension was dropped onto a carbon-coated copper grid (400 mesh) and stained with a 2% phosphotungstic acid solution. The TEM images were collected with a charge-coupled device camera (XR16, AMT Imaging, Woburn, MA, USA) at an exposure time of 3.2 s.

### SAXS and data analysis

SAXS was conducted at beamline BL15A2 at the Photon Factory (High Energy Accelerator Research Organization, Tsukuba, Japan).^31^ The liposome suspension is introduced into the COP device fixed on a beamline stage using syringe pumps (Fig. 1(b)). The LNP suspension is introduced into the center of the microchannel, and saline or ethanol is introduced into the side channels to generate a sheath flow. The total flow rate is 50 μL min^−1^ at a FRR of 5, and the SAXS data are obtained at a wavelength of 1.213 Å, with the SAXS detector (PILATUS3 X 2M, DECTRIS, Baden, Switzerland) distance set to 1.5 m. For time-resolved SAXS, the sample stage is moved in the height direction with steps of 2 mm. SAXS images of each measurement point are collected with exposure times of 1 s, and 60 images are integrated to generate the SAXS profile since no effect of the radiation damage was observed. SAXS data were analyzed using SAngler.^32^

### MD simulations

MD simulations were conducted using the LAMMPS software (Sandia National Laboratories, Albuquerque, NM, USA),^33^ and the SPICA force field was employed for the POPC bilayer systems containing ethanol.^34–36^ The CG representation of each molecule is shown in Fig. S1.† Ethanol represented as a single CG site is introduced as an amino acid side chain analogue in the SPICA force field.^37^ We set up lamellar membrane systems with different ethanol concentrations. For each simulated system, a short MD run using an NVT ensemble was conducted to thermalize the system for 100 ps after energy minimization. Subsequently, 1.1 μs NPT MD production runs were conducted, and the trajectories in the last 100 ns were used in the structural analyses. The temperature was controlled at 310 K using the Nosé–Hoover thermostat,^38,39^ and the pressure was controlled at 1 atm using the Parrinello–Rahman semi-isotropic coupling barostat.^40,41^ The Lennard-Jones-type interaction for a nonbonding interaction was simply truncated at 1.5 nm, although the coulombic interactions among the lipid headgroups were calculated *via* the P^3^M method.^42^ The size of the time step used to integrate the equation of motion was 10 fs.

The structure factors of the lamellar systems are calculated as
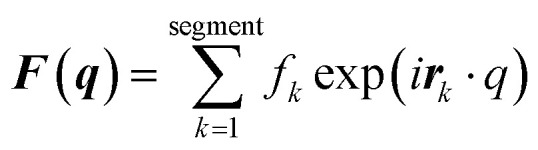
where *f*_*k*_ is the atomic scattering factor of the *k*-th CG segment, which is neglected and assumed to be 1 in this study. The scattering intensity *I*(*q*) is then obtained using the structure factor and polarization factor *P*:*I*(*q*) = |***F***(***q***)|^2^*P*,
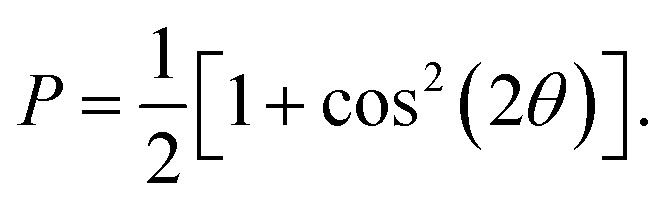


The calculated segmental order parameters of the lipid tails are defined as
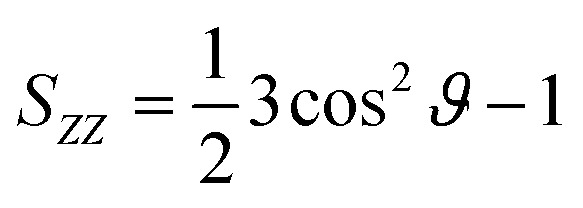
where *ϑ* is the angle of the bond vector connecting the adjacent segments in the hydrophobic tail with respect to the bilayer normal.

## Author contributions

Conceptualization: M. M. and M. T.; data curation: M. M, N. K., Y. O., K. S., Y. M., and W. S.; formal analysis: M. M, N. K., Y. O, K. S., K. S., Y. M., A. I., H. T., K. Y., N. S., and W. S.; methodology: M. M., K. S., Y. M., and N. S.; investigation: M. M., N. K., Y. O., K. S., Y. M., and W. S.; writing-original draft: M. M.; writing-review and editing: M. M., W. S., and M. T.; funding acquisition: M. M., N. K., N. S., W. S., and M. T.; resources: M. M., K. Y., N. S., and W. S.; supervision: M. M. and M. T.

## Conflicts of interest

There are no conflicts to declare.

## Supplementary Material

NA-006-D3NA01073B-s001
