# Supplementary material for: Understanding the effects of ethanol on the liposome bilayer structure using microfluidic-based time-resolved small-angle X-ray scattering and molecular dynamics simulations
Source: Nanoscale Adv. 2024 Mar 25;6(8):2166–76. doi: 10.1039/d3na01073b (PMC11019499; doi:10.1039/d3na01073b)
Supplement: NA-006-D3NA01073B-s001 [file NA-006-D3NA01073B-s001.pdf]

Electronic Supplementary Material (ESI) for Nanoscale Advances.
This journal is © The Royal Society of Chemistry 2024

Supporting Information

Understanding the effects of ethanol on structural changes in liposomes using microfluidic-based time-resolved small-angle X-ray scattering and MD simulations

Masatoshi Maeki,[a,b,c] Niko Kimura,[d,#1] Yuto Okada,[d] Kazuki Shimizu,[d] Kana Shibata,[e] Yusuke Miyazaki,[f] Akihiko Ishida,[a] Kento Yonezawa,[c,#2] Nobutaka Shimizu,[c] Wataru Shinoda,[f] and Manabu Tokeshi[a*]*

[a] *Division of Applied Chemistry, Faculty of Engineering, Hokkaido University, Kita 13 Nishi 8, Kita-ku, Sapporo 060-8628, Japan*

[b] *JST PRESTO, 4-1-8 Honcho, Kawaguchi, Saitama 332-0012, Japan*

[c] *Institute of Materials Structure Science, High Energy Accelerator Research Organization (KEK), Tsukuba, Ibaraki 305-0801, Japan*

[d] *Graduate School of Chemical Sciences and Engineering, Hokkaido University, Kita 13 Nishi 8, Kita-ku, Sapporo 060-8628, Japan*

[e] *Department of Materials Chemistry, Nagoya University, Chikusa-ku, Nagoya 464-8603, Japan*

[f] *Research Institute for Interdisciplinary Science, Okayama University, Okayama 700-8530, Japan*

[#1] *Present address: Department of Applied Chemistry, Faculty of Engineering, Kyushu University*

[#2] *Present address: Center for Digital Green-innovation, Nara Institute of Science and Technology, Ikoma, Nara 630-0192, Japan*

Corresponding authors:

Masatoshi Maeki

E-mail address: m.maeki@eng.hokudai.ac.jp

Tel: +81-11-706-6773 Fax: +81-11-706-6745

Manabu Tokeshi

E-mail address: tokeshi@eng.hokudai.ac.jp

Tel: +81-11-706-6744 Fax: +81-11-706-6745

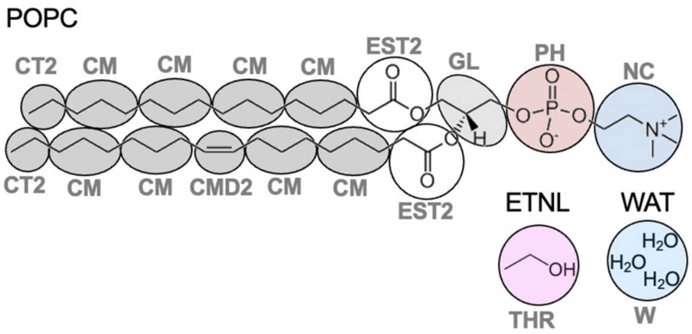

Figure S1: Definition of each coarse-grained (CG) site in a SPICA force field. The molecular and CG-site names are given in black and gray, respectively.

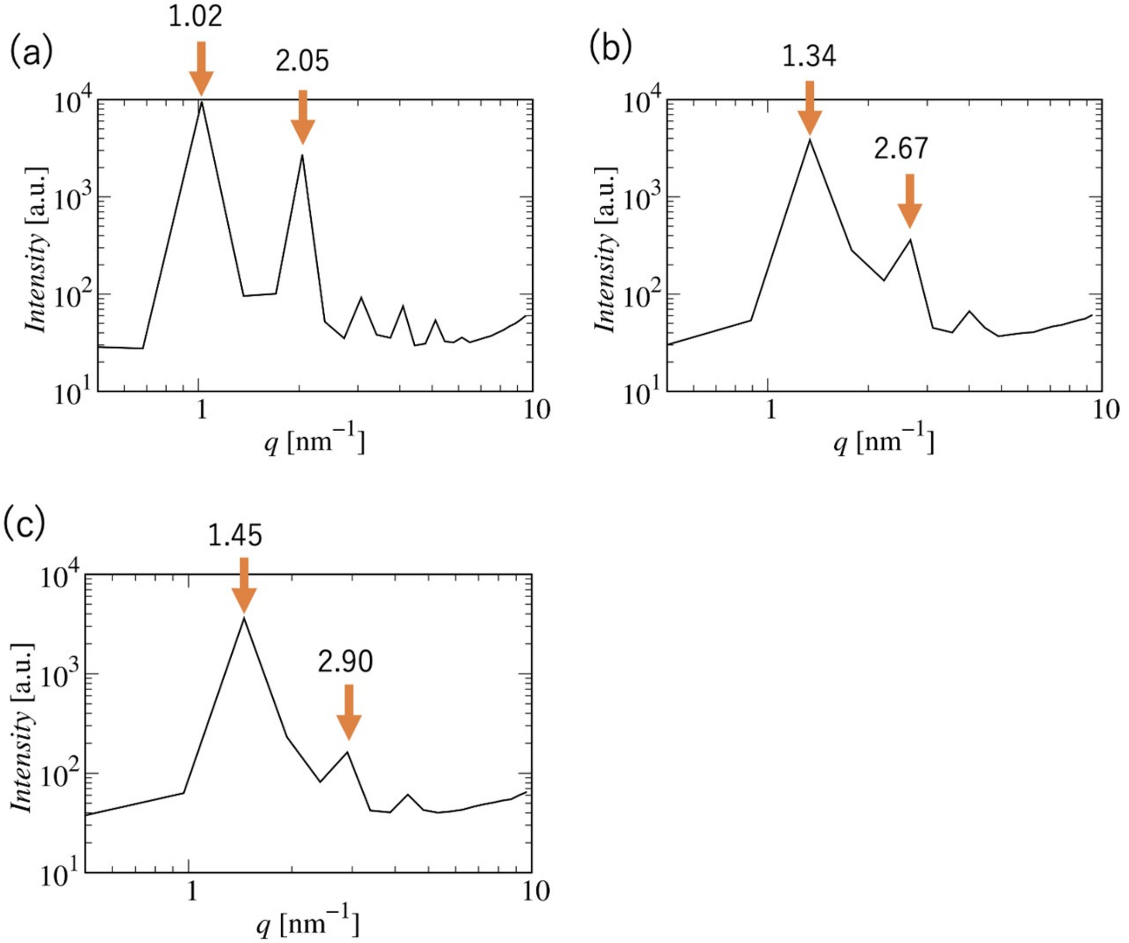

Figure S2: Structure factors of Systems (a) 1, (b) 2, and (c) 3. The arrows represent the positions of the first and second peaks, with their q-values.

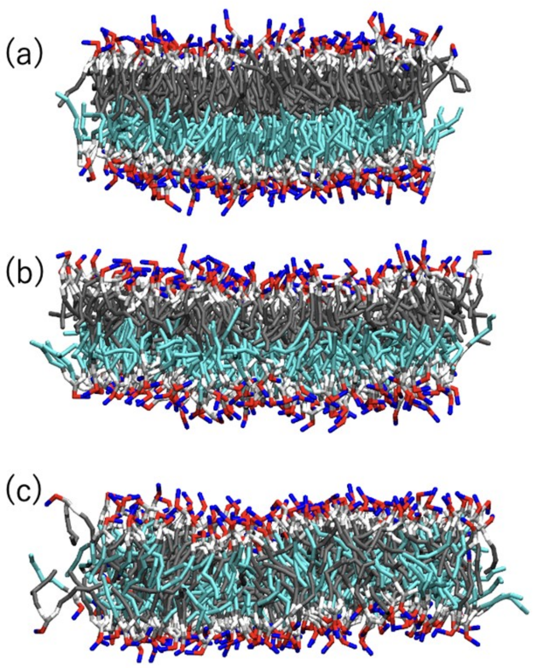

Figure S3: Final snapshot of Systems (a) 1, (b) 2, and (c) 3. The color code is the same as that in Figure 6, except for the hydrocarbons of the initial lower lipid layer, which is in cyan to elucidate the interlayer coupling and lipid exchange between the upper and lower layers (flip-flop).
